# Combining Intensive Rehabilitation With a Nonfunctional Isokinetic Strengthening Program in Adolescents With Cerebral Palsy: Protocol for a Randomized Controlled Trial

**DOI:** 10.2196/43221

**Published:** 2023-05-03

**Authors:** Mathias Guérin, Benoit Sijobert, Benjamin Zaragoza, Flore Cambon, Laurence Boyer, Karine Patte

**Affiliations:** 1 Unité de rééducation Institut Saint-Pierre Palavas-les-Flots France

**Keywords:** isokinetic, cerebral palsy, gait analysis, spasticity, muscle strength

## Abstract

**Background:**

Cerebral palsy is the most common brain injury in the pediatric population. Patients with cerebral palsy present different affectations such as decreased muscle strength, gait deviations, impaired proprioception, and spasticity. Isokinetic strengthening programs combined with intensive rehabilitation may improve muscle strength and therefore gait efficiency.

**Objective:**

The primary aim of this randomized controlled trial is to compare the effect of an intensive rehabilitation combined with a nonfunctional isokinetic progressive strengthening program to an intensive rehabilitation alone on gait parameters and muscle strength in patients with cerebral palsy. Another goal of this study is to determine whether adding an isokinetic program to intensive rehabilitation is more effective than intensive rehabilitation alone at decreasing spasticity and improving joint position sense in patients with cerebral palsy.

**Methods:**

A total of 30 adolescents with spastic diplegia cerebral palsy (Gross Motor Function Classification System levels I to III) will be randomized, by an independent researcher, into a 3-week intensive rehabilitation and isokinetic progressive strengthening group or an intensive rehabilitation control group. Gait parameters, muscle strength, spasticity, and knee joint position sense will be assessed. These variables will be evaluated at baseline (T0) and at the end of the intervention (T1). The intensive rehabilitation will consist of physiotherapy sessions twice a day and hydrotherapy and virtual reality gait training once a day. The isokinetic training group will have a total of 9 supervised isokinetic strength training sessions focusing on knee flexors and extensors with different execution speeds.

**Results:**

The protocol has been accepted by the French National Ethics Committee in October 2022. The inclusion of patients will start in November 2022.

**Conclusions:**

The combination of intensive rehabilitation with an isokinetic program on knee flexors and extensors has not been studied yet. The findings of this study may determine if an isokinetic strength training program of knee flexors and extensors is beneficial for the improvement of gait parameters, muscle strength, spasticity, and joint position sense in adolescents with spastic diplegia.

**International Registered Report Identifier (IRRID):**

PRR1-10.2196/43221

## Introduction

### Background

Cerebral palsy is considered the most common brain injury in the pediatric population [1]. Motor impairments are commonly observed in adolescents with cerebral palsy [2]. Sensory impairments, cognitive disorders, epilepsy, and nutritional deficiencies are also often found in patients with cerebral palsy [2]. Cerebral palsy prevalence ranges from 1.5 to 2.5 cases per 1000 births and does not differ within different western countries [3]. Spasticity is regularly described as the most common motor impairment in the cerebral palsy population and is characterized by an increased stretch reflex. Spasticity is movement dependent, increasing with a higher velocity [4]. Spasticity is often measured through an isokinetic dynamometer to passively stretch the targeted muscles [4-6].

Muscle strength is commonly decreased in adolescents with cerebral palsy. Quadriceps and hamstring muscles are important to perform daily activities such as standing, walking, and climbing stairs [7]. Knee extensor strength is described as a good indicator of total lower limb muscle strength. Isokinetic dynamometry is the gold standard to measure muscle strength [6]. Isokinetic dynamometry has been described as a reliable tool to measure quadriceps and hamstring muscle strength in children with cerebral palsy [6]. Isokinetic dynamometry can also be used within a reeducation program to increase muscle strength in children with cerebral palsy [6]. An isokinetic strength training program focusing on knee flexors and extensors has shown some promising results in improving muscle strength in children with cerebral palsy [8]. MacPhail and Kramer [8] did not find any improvement in gait parameters in patients with cerebral palsy after an isokinetic program focusing on knee flexors and extensors. However, they did not implement such a program within an intensive rehabilitation program. A recent systematic review has shown that an isokinetic strength training program focusing on knee flexors and extensors improves muscle strength, mobility, and gait in patients with stroke [9]. Functional power training has been shown to be effective training to improve muscle strength, walking capacities, and resting somatosensory cortical activity in children with cerebral palsy [10-12]. Power training exercises such as walking, sit-to-stand, and side-walking should form part of an intensive rehabilitation program. Medium-speed concentric and eccentric isokinetic training is considered strength training, whereas low-speed isokinetic training produces hypertrophy [13]. Finally, high-speed isokinetic training is described as power training [13].

Gait deviations are frequently observed in adolescents with cerebral palsy due to motor impairment. Gait speed, stride length, step width, and stance time are important factors for an effective gait [14,15]. Lower limb muscle fatigue is commonly observed in patients with cerebral palsy and drastically affects their gait abilities and distances [16,17]. Usually, patients with cerebral palsy receive physiotherapy treatment for long periods; however, decreased muscle strength of the lower limbs has been shown to be associated with impaired gait function in children with cerebral palsy. Therefore, treatment for patients with cerebral palsy should focus on strengthening lower limb muscles to improve gait parameters [15,18].

Proprioception has been defined as the ability of an individual to integrate sensory signals from mechanoreceptors, allowing him to determine the position of a joint in space and its movement [19]. Knee proprioception is provided by afferent signals that are located inside or around the joint [19,20]. Its functions are to protect the knee joint against excessive injurious movement, stabilize the knee during static postures, and coordinate complex movements [19-21]. Wingert et al [22] have shown that children with cerebral palsy who present with gait disturbances have poorer proprioception than typically developing children. Over the last decade, 2 different proprioception assessment techniques have been described in the literature [21]: joint position sense and joint motion sense. Strength training has been described as an effective intervention to improve proprioception in children with cerebral palsy [23].

Intensive rehabilitation has been shown to be an effective program to improve functional motor outcomes in children with cerebral palsy [7,24-26]. A recent practice guideline to improve physical function in children with cerebral palsy has shown that a high enough dose of practice is required in order to improve motor abilities [26]. Jackman et al [26] have shown that intensive blocks of therapy were better than low-dose regular therapy in order to improve motor abilities in patients with cerebral palsy. Elgawish and Zakaria [27] have shown that intensive physiotherapy is more effective than standard physiotherapy in improving gross motor function in children with spastic diplegia. Bleyenheuft et al [24,25] have shown that an intensive training protocol including upper and lower extremities was effective in improving lower extremity function in children with unilateral and bilateral spastic cerebral palsy. In a meta-analysis, Moreau et al [28] have shown that gait training is an important component of rehabilitation to improve gait parameters, such as gait speed, in children with cerebral palsy. In their clinical guideline, Jackman et al [26] have shown that 30 to 40 hours of practice are required to improve motor abilities. Combining intensive rehabilitation with an isokinetic strengthening program has not been studied yet.

### Objectives

The primary aim of this randomized controlled trial is to compare the effect of intensive rehabilitation combined with an isokinetic progressive strengthening program to intensive rehabilitation alone on gait parameters and muscle strength in patients with cerebral palsy. Another goal of this study is to determine whether adding an isokinetic program is more effective than just intensive rehabilitation in decreasing spasticity and improving joint position sense in patients with cerebral palsy.

## Methods

### Study Design

This study will be a prospective randomized controlled trial. Adolescents with spastic diplegia cerebral palsy will be randomized by an independent researcher through a computer-based method into an isokinetic training group (IG) or a control group (CG). The block randomization method will be used to keep the number of subjects in each group equal. The IG will receive a 3-week isokinetic strengthening program together with intensive rehabilitation, and the CG will only follow the intensive rehabilitation. Patients will not be informed if they are part of the intervention or the CG. Outcome measures will be done at baseline (T0) and at the end of the intervention (T1). The protocol has been accepted by the French National Ethics Committee (IDRCB: 2022-A00431-42).

### Sample Size

Sample size calculation was realized using G*POWER statistical programming (version 3.1.9.2; Franz Faul): power (1−α error *P*)=.85, α=.05, effect size=1.1, with a 2-tailed for a comparison of 2 independent groups. This calculation was based on the results of a pilot study based on ratio improvement following 2 specific interventions in patients with cerebral palsy. It will take 15 participants in each group to complete this study.

### Participants

A total of 30 pediatric patients will be recruited from the Institut St Pierre, France. The inclusion criteria for this study will be adolescents aged from 11 up to 18 years with a diagnosed spastic diplegia cerebral palsy, patients with walking abilities (Gross Motor Function Classification System levels I to III), the ability to activate knee flexors and extensors voluntarily, and cognitive abilities to understand the instructions. The exclusion criteria for this study will be cardiovascular disorders; fractures of any type; history of hip subluxation, dislocation, or important scoliosis (>40° of curvature); surgery of the lower limb within the last year; botulinum toxin injection within the last 3 months; significant contracture of the knee flexors and extensors (less than 90° of knee active range of motion [ROM]); and insufficient understanding of the French language. All the participants and their legal tutor will be informed of the study procedures in agreement with the ethical standards of the Helsinki Declaration and will sign an informed consent.

### Intensive Rehabilitation

**Figure 1:**
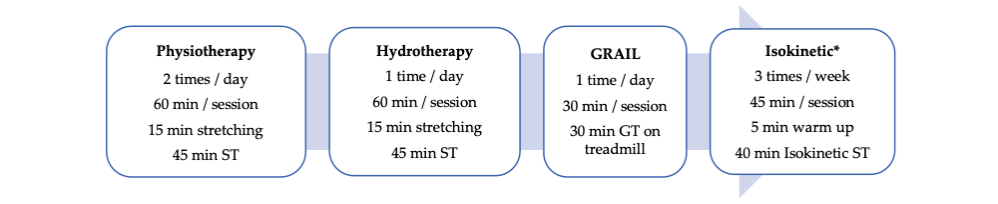
Rehabilitation program with the total amount of training. During the 3 weeks of rehabilitation, the isokinetic training group and control group will respectively perform a total of 48 hours and 41 hours of active training. *Only performed by the isokinetic training group. GRAIL: Gait Real-time Analysis Interactive Lab; GT: gait training; ST: strength training.

The intensive rehabilitation will be conducted over a 3-week period. All participants will follow the intensive rehabilitation. Physiotherapy will be performed twice a day. Hydrotherapy and virtual reality gait training will be performed on a daily basis. During the 3 weeks of rehabilitation, the IG and CG will practice for a total of 59 and 52 hours, respectively. The hourly repartition can be found in Figure 1.

**Figure 2:**
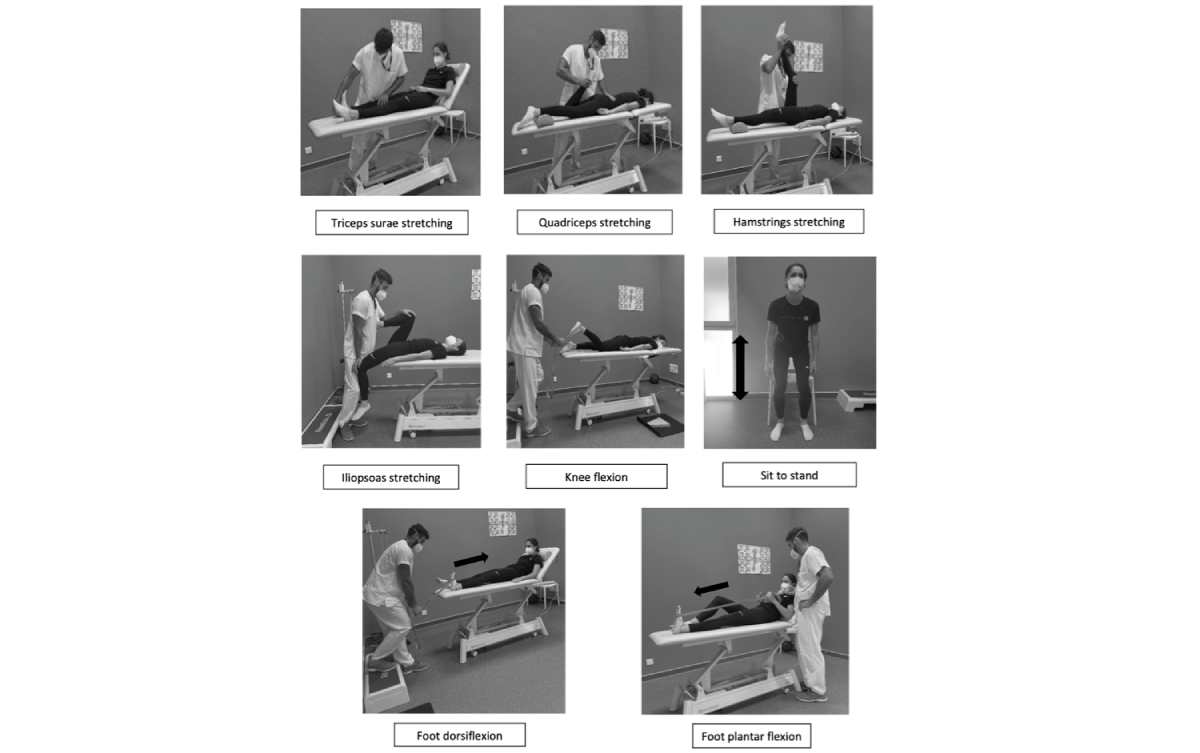
Images of the guidelines for the intensive rehabilitation program and the isokinetic strength training program. Both groups will follow the exact same intensive rehabilitation program. Only the isokinetic training group will follow the isokinetic strength training program. *Knee flexors and extensors are trained at various speeds in concentric and eccentric modes with different intensities depending on the training week.

Physiotherapy sessions are expected to last for 60 minutes and will include lower limb stretching exercises and strengthening of the lower extremities (Table 1). All exercises realized during a typical physiotherapy session are described in Table 2. Hydrotherapy sessions will last for 60 minutes and will include stretching exercises, aerobic exercises, and strengthening of the lower extremities (Table 3). Hydrotherapy exercises are realized with water up to the patient’s belly. All exercises realized during a typical hydrotherapy session are described in Table 2. The virtual reality gait training will last for 30 minutes in total and will focus on improving different gait parameters with video games. Another aim of the virtual reality training will be to perform balance exercises. Perturbations will be added to challenge participants. All exercises realized during a Gait Real-time Analysis Interactive Lab (GRAIL) session are described in Table 2. The same video games will be performed for every adolescent (Table 4). A summary of all exercises performed during the intensive rehabilitation is presented in Figure 2.

**Table 1:** Characteristics and rehabilitation volume for the physiotherapy sessions.

Interventions and exercises		Sets, n	Repetitions (seconds), n	Contraction types
**Stretching**				
	Triceps surae	2	20	N/A^a^
	Quadriceps	2	20	N/A
	Hamstrings	2	20	N/A
	Iliopsoas	2	20	N/A
**Strengthening**				
	Foot plantar flexion with elastic band	6	10	CON^b^ and ECC^c^
	Foot dorsiflexion with elastic band	6	10	CON and ECC
	Sit to stand	6	10	CON and ECC
	Knee flexion with elastic band	6	10	CON and ECC

^a^N/A: not applicable.

^b^CON: concentric realized as fast as possible.

^c^ECC: eccentric maintained for 2-3 seconds.

**Table 2:** Description of intensive rehabilitation sessions performed by adolescent in both groups.

Interventions and exercises				Description		Resistance added
**Physiotherapy**						
	**Stretching**					
		Triceps surae	Patient in supine position. PT^a^ is pushing the patient foot in dorsiflexion with the knee extended as much as possible		N/A^b^	
		Quadriceps	Patient in prone position. PT is bending the patient knee and maintaining the patient hip against the table		N/A	
		Hamstrings	Patient in supine position. PT is maintaining the patient knee extended and adding as much hip flexion as possible. The opposite leg must stay extended on the table		N/A	
		Iliopsoas	Patient in supine position on the lower part of the table. PT is maintaining a hip extension with a knee flexion. The opposite leg is placed on the chest of the PT		N/A	
	**Strengthening**					
		Foot plantar flexion	Patient in supine position with knee extended. Ankle placed in dorsiflexion. Elastic band placed around the ball of the foot and help by the patient		Resistance band of various resistances	
		Foot dorsiflexion	Patient in supine position with knee extended. Ankle placed in plantar flexion. Elastic band placed around the instep of the foot and held by the PT		Resistance band of various resistances	
		Sit to stand	Patient seating on a chair and push with his feet on the ground to stand		Dumbbells from 5 kg to 10 kg	
		Knee flexion	Patient in prone position with knee extended. Elastic band is placed around patient ankle and held by the PT		Resistance band of various resistances	
**Hydrotherapy**						
	**Stretching**					
		Triceps surae	Patient standing against the pool. One lower limb in front of the other. Both feet on the ground with the back foot in maximal dorsiflexion		N/A	
		Quadriceps	Patient standing against the pool. Holding one lower limb and bending toward knee flexion while the other hand is stabilizing against the pool.		N/A	
		Iliopsoas	Patient kneeling against the pool. One knee against the ground and the other one bended. Pushing his gravity center forward		N/A	
	**Strengthening**					
		Forward walking	Patient standing and walking forward at high intensity inside the swimming pool with water up to the belly.		Resistance band	
		Backward walking	Patient standing and walking backward at high intensity inside the swimming pool with water up to the belly.		Resistance band	
		Side walking	Patient standing and walking from side to side at high intensity inside the swimming pool with water up to the belly		Resistance band	
		Calf raises	Patient standing against the pool with water up to the belly. Pushing against the ground toward maximal plantar flexion and going back to the ground slowly		Water resistant weight	
		Squat	Patient standing against the pool with water up to the belly. Squatting up to 90 degrees of knee flexion and back to the initial position		Water resistant weight	
**GRAIL^c^**						
	**Gait training**					
		Rope bridge	Patient standing on a treadmill. 4 markers are placed on posterior and anterior superior iliac spine. A self-paced mode is used to let the patient walk at a comfortable speed		Sway: level 1 to 3; pitch: level 1 to 3; encounters: level 1 to 3	
		Step on it	Patient standing on a treadmill. 2 markers are placed on both lateral malleoli. Treadmill speed is set based on gait analysis assessment.		Increasing step length and decreasing step width	
	**Balance**					
		Perturb	Patient is standing on the treadmill. Anteroposterior and mediolateral stimuli are produced from the treadmill.		Automatic mode starting from level 1 to 5	

^a^PT: physiotherapist.

^b^N/A: not applicable.

^c^GRAIL: Gait Real-time Analysis Interactive Lab.

**Table 3:** Characteristics and rehabilitation volume for the hydrotherapy sessions.

Interventions and exercises			Sets, n		Repetitions (seconds), n		Contraction types
**Stretching**							
	Triceps surae	2		20		N/A^a^	
	Quadriceps	2		20		N/A	
	Iliopsoas	2		20		N/A	
**Strengthening**							
	Walking forward	6		30		N/A	
	Walking backward	6		30		N/A	
	Side walking	6		30		N/A	
	Calf raises	5		10		CON^b^ and ECC^c^	
	Squats	5		10		CON and ECC	

^a^N/A: not applicable.

^b^CON: concentric realized as fast as possible.

^c^ECC: eccentric maintained for 2-3 seconds.

**Table 4:** Characteristics and rehabilitation volume for the Gait Real-time Analysis Interactive Lab sessions.

Exercises	Aim	Sets, n	Duration (minutes^a^), n	Parameters
Rope bridge	Walk on treadmill with perturbations	4	2	Sway, pitch, and encounters
Perturb	Stand on the treadmill with perturbation to improve balance	4	2	Automatic mode
Step on it	Walk on the treadmill with targets to hit to improve step width and length	4	2	N/A^b^

^a^Minutes for the time for each exercise; seating breaks of 1 minute are respected between each sets.

^b^N/A: not applicable.

### Isokinetic Training Intervention

The IG will also receive a total of 9 supervised isokinetic training sessions over 3 weeks based on given guidelines for isokinetic training [29-31]. Adolescents will train between 70% and 80% of the maximal value in Newton-meter reached during the test at the different speeds (Table 5). A visual feedback will be displayed to the participants, with a frame range showing the target strength to reach for every set. A rest time of at least 24 hours between sessions will be observed for every participant. Based on MacArdle et al’s [13] findings, 1 single training session will consist of 3 sets of 5 repetitions at a speed of 30 degrees/s concentrically, 3 sets of 8 repetitions at 120 degrees/s concentrically, and 3 sets of 3 repetitions at 30 degrees/s eccentrically. Therefore, strength, hypertrophy, and power will be performed. One repetition consisted of a complete knee extension and flexion, based on the ROM reached during the first assessment. A 90-second rest will be respected between each set. Every session will be preceded by a 5-minute warm-up and followed by a 5-minute cooldown. All exercises realized during isokinetic sessions are shown in Figure 2. One session is expected to last for approximately 45 minutes [29,30,32]. A summary of the exercises performed in the isokinetic strengthening program is presented in Figure 2.

**Table 5:** Isokinetic strengthening program for the intervention group.

Weeks	Sets, n	Intensity, %^a^	Contraction type
1 and 2	3	70	CON^b^ and ECC^c^
3 and 4	3	75	CON and ECC
5 and 6	3	80	CON and ECC

^a^Percentage from maximal reached value at each speed.

^b^CON: concentric.

^c^ECC: eccentric.

### Primary Outcomes

#### Strength

Muscle strength will be assessed with an isokinetic dynamometer (CON-TREX, MJ Multijoint module, Medimex, CMV AG). Patients will be installed as described previously. Patients will perform an aerobic warm-up for 5 minutes and stretching of the quadriceps and hamstring prior to the test. The ROM will be set based on the extension abilities of the patients. Knee flexors and extensors will be assessed in a concentric mode for 3 repetitions at a speed of 30 degrees/s, 5 repetitions at a speed of 120 degrees/s, and for 8 repetitions at a speed of 180 degrees/s; in an isometric mode at 45° of knee flexion for 5 seconds; and in an eccentric mode for 3 repetitions at a speed of 30 degrees/s. The isokinetic equipment is shown in Figure 2.

Between the 2 concentric, the isometric, and the eccentric tests, 90 seconds of recovery will be allowed. Verbal encouragement will be provided for all participants during the entire test. The highest value in Newton-meter, the maximum reached speed in degrees/s, and the total work produced in joules will be considered for data analysis.

A recent meta-analysis from Muñoz-Bermejo et al [6] showed good to excellent levels of reliability of isokinetic dynamometer in measuring knee extensors and knee flexors strength in children with cerebral palsy, with an interclass correlation coefficient (ICC) ranging from 0.7 to 1 in the dominant and the nondominant lower limb.

#### Gait Assessment

Patients will perform a walking test on the GRAIL (Motekforce Link). The GRAIL technology consisted of a split-belt treadmill with integrated force plates, an incurved virtual reality screen, and a 3D motion capture system (VICON). The treadmill is built with a self-paced mode, allowing patients to walk at a comfortable speed during the entire test.

Spatiotemporal parameters such as gait speed, stride length, step width, and stance time will be recorded during the walking test. Gait speed (m/s) is defined as the average speed recorded during the entire test after removing the first and last seconds to undo the acceleration and deceleration phenomena. Stride length (cm) is defined as the average anteroposterior distance between heel strikes of the same lower limb. Step width (cm) is defined as the average mediolateral distance between both feet. Stance time (s) is defined as the time between heel strike and toe-off from the same lower limb.

Kinematics data will also be collected through the 3D motion capture system. A total of 22 markers will be placed on patients’ lower limbs following the human body model, which has been described as a valid method to measure kinematics [33]. Hip, knee, and ankle ROM will be captured during the entire gait assessment in the sagittal, transversal, and horizontal planes. Electromyography activity will be recorded for both lower limbs using surface electrodes on 2 important muscles. The rectus femoris and biceps femoris will be recorded. These 2 muscles are important for knee function, including extension and flexion.

A 6-minute walking test (6MWT) will also be performed on the GRAIL. The treadmill will be set to the self-paced mode. Patients will be asked to walk during the entire test. The total distance reached during the test will be registered for data analysis. Performing the 6MWT on the GRAIL technology has shown good construct validity and reproducibility [34,35]. The Borg scale will also be measured before and after the 6MWT for every patient as an indicator of the level of exertion perceived during the test [34,35].

### Secondary Outcomes

#### Spasticity

Spasticity within knee flexors and extensors will be assessed with an isokinetic dynamometer (CON-TREX, MJ Multijoint module, Medimex, CMV AG) by applying a passive movement at a speed of 180 degrees/s to stretch the muscles. Patients will be installed on the isokinetic seat with 80° of hip flexion, 90° of knee flexion, and no restriction within the ankle joint. The mechanical axis of the dynamometer will be aligned with the lateral epicondyle of the femur. The tibial pad will be placed 3 cm above the lateral malleolus. The trunk and the thigh of the patients will be stabilized with belts. Both limbs will be assessed for 10 consecutive times from 90° to 25° of knee flexion and back to 90°. A 60-second rest break will be respected between each movement. The patient will receive the instruction to rest as much as possible during the entire movement. Prior to the spasticity test, a filter for gravity correction will be applied based on the patient’s limb weight. The isokinetic dynamometer will record the forces, angles, and speed during the entire movement. The first and last 5° of each movement will be excluded from the data analysis to avoid errors from the effect of inertia [24]. The test will be interrupted if the force, in Newton, applied to the dynamometer is greater than half the patient’s body weight. Patients will be instructed to stop the test if they feel any pain or are scared [36].

The peak resistive torque will be calculated at baseline and after a 3-week intensive training program within both groups. In the knee, the peak resistive torque has shown good relative reliability at a speed of 180 degrees/s, with an ICC of 0.86 for knee extensor peak torque and an ICC of 0.80 for knee flexor peak torque [36].

#### Knee Joint Position Sense

Knee JPS will be assessed with an isokinetic dynamometer (CON-TREX, MJ Multijoint module, Medimex, CMV AG). Patients will be installed as described previously. The patient’s resting position will be set to a preselected joint angle of 45° of knee flexion. Knee JPS will consist of the position-reposition angle difference of the dominant leg. The angular speed for the repositioning task will be set at 2° per seconds as described by El Shemy [37]. Prior to the test, every adolescent will participate in a practice session to become familiar with the task. For the test, the patient’s limb will first be placed at a knee angle of 45° for 10 seconds, and then the lower limb will go back to 90° of knee flexion. Finally, the dynamometer will move the lower limb through extension at the same speed. Once patients feel that they have reached the target position again, they will press the button and stop the test. Patients will be blindfolded, and the test will be performed in quiet surroundings to eliminate visual and auditory stimuli. The test will be performed 3 consecutive times, and the mean will be calculated for data analysis.

### Data Analysis

Descriptive statistics will be used to describe gender, age, and Gross Motor Function Classification System. Primary and secondary outcomes will be analyzed in the same way. The assumption of normality will be checked by visual inspection of the q-q plot and the box plot of the data within groups. A Shapiro-Wilks test will also be performed on the data within groups. The homogeneity of variance will be checked using Levene test.

If the data are normally distributed, an independent *t* test will be carried out for each variable to see if there are differences between the experimental and control groups. The 95% CI for the mean differences will be determined. Pearson *r* will be used to calculate the effect size.

If the data are not normally distributed, a Wilcoxon rank-sum test will be used for each variable to determine if there are any significant differences between groups. Pearson *r* will be calculated as a measure of effect size.

The data analysis will be performed using SPSS (25.00, IBM Corp). For the data analysis, the researchers will use a 95% CI, considering all those variables with a *P* value of less than .05 to be statistically significant.

### Ethics Approval

This research is promoted by the Institut St-Pierre, France. A parent or legal tutor together, with participants, will fill out a written consent. All participants will join this study voluntarily and will be informed that they can stop their participation at any time. Full data will be available only to members of the research team, and anonymization of the data will be done prior to publication. Written observation reports will be registered during the entire trial by the investigators. Adverse events will be reported to the medical practitioner and the trial sponsor. Unexpected serious adverse events will be transferred to the ethical committee within 15 days. Trial results will be published in academic journals and presented at conferences worldwide. Participants, their families, and health care professionals will have the option to receive a summary of the study results. The study will be conducted in accordance with the Declaration of Helsinki, and the protocol has been approved by the Comité de protection des personnes Sud-Méditerranée II (CPP 222 B21) IDRCB 2022-A01790-43. The date of approval was October 3, 2022.

## Results

The protocol was accepted by the French National Ethics Committee in October 2022. The inclusion of patients will start in November 2022. Data collection is expected to be completed in June 2024. The results of the trial are expected to be available by the end of 2024.

## Discussion

### Principal Findings

This study will evaluate the effects of intensive rehabilitation combined with an isokinetic progressive strengthening program on gait parameters, muscle strength, spasticity, and joint position sense. Both groups will receive the same intensive rehabilitation, and the intervention group will also have an isokinetic program. The findings of this study may determine if an isokinetic strength training program of knee flexors and extensors is beneficial to improve gait parameters, muscle strength, spasticity, and joint position sense in adolescents with spastic diplegia. Positive findings are expected for this clinical trial. If this is the case, isokinetic strength training programs should be considered as a complementary intervention for patients with cerebral palsy.

It has been decided to measure and strengthen knee flexors and extensors, as they have been described as good indicators for total lower limb muscle strength [6,38]. Strength training programs have been designed and tested on children with spastic diplegia [30,39,40]. Aye et al [40] have shown an improvement in muscle strength, gait parameters, and gross motor function measure after a strength training program for hip and knee extensors. In their study, MacPhail and Kramer [8] have shown that an isokinetic strength training program focusing on knee flexors and extensors could significantly improve strength in patients with cerebral palsy. MacPhail and Kramer [8] did not find significant improvement in gait parameters following an isokinetic strength training program. However, they did not implement such a program within an intensive rehabilitation program.

### Limitations

The intensive rehabilitation program could not be longer than 3 weeks due to school, and a longer period of time might be important to improve gait functions and muscle strength in patients with cerebral palsy.

The total amount of training was not kept equal for both groups due to the isokinetic training program, and this might have an influence on the results.

### Conclusions

Nowadays, there is missing information about the effects of an intensive rehabilitation program combined with an isokinetic strength training program on gait parameters. The combination of intensive rehabilitation with an isokinetic program on knee flexors and extensors has not been studied yet. Positive findings of this study may suggest that an isokinetic strength training program should be implemented within the traditional treatment of children with spastic diplegia.

## Appendix

Multimedia Appendix 1 CONSORT-eHEALTH checklist (V 1.6.2).

## Abbreviations

6MWT: 6-minute walking test
CG: control group
GRAIL: Gait Real-time Analysis Interactive Lab
ICC: interclass correlation coefficient
IG: isokinetic training group
ROM: range of motion

## Data Availability

The data are not publicly available, as they have not been collected yet. The data will be uploaded to publish this paper.
