# Supplementary material for: Combining Intensive Rehabilitation With a Nonfunctional Isokinetic Strengthening Program in Adolescents With Cerebral Palsy: Protocol for a Randomized Controlled Trial
Source: JMIR Res Protoc. 2023 May 3;12:e43221. doi: 10.2196/43221 (PMC10193213; doi:10.2196/43221)
Supplement: Multimedia Appendix 1 [file resprot_v12i1e43221_app1.pdf]

CONSORT-EHEALTH Checklist V1.6.2 Report	Manuscript Number	43221
**(based on CONSORT-EHEALTH V1.6), available at [http://tinyurl.com/consort-ehealth-v1-6].**		
**Date completed**		
3/6/2023 4:56:24		
**by**		
Mathias Guerin		
TITLE		
**1a-i) Identify the mode of delivery in the title**		
**1a-ii) Non-web-based components or important co-interventions in title**		
The article is not a internet based intervention		
**1a-iii) Primary condition or target group in the title**		
ABSTRACT		
**1b-i) Key features/functionalities/components of the intervention and comparator in the METHODS section of the ABSTRACT**		
**1b-ii) Level of human involvement in the METHODS section of the ABSTRACT**		
Difference between groups and outcomes measured are well explained in the abstract		
**1b-iii) Open vs. closed, web-based (self-assessment) vs. face-to-face assessments in the METHODS section of the ABSTRACT**		
the physiotherapist that is together with the children		
**1b-iv) RESULTS section in abstract must contain use data**		
Face to face by a clinician		
**1b-v) CONCLUSIONS/DISCUSSION in abstract for negative trials**		
No results are available as this is a research protocol		
INTRODUCTION		
**2a-i) Problem and the type of system/solution**		
**2a-ii) Scientific background, rationale: What is known about the (type of) system**		
explanation of the affectation from cerebral palsy (CP)		
METHODS		
**3a) CONSORT: Description of trial design (such as parallel, factorial) including allocation ratio**		
**3b) CONSORT: Important changes to methods after trial commencement (such as eligibility criteria), with reasons**		
primary and secondary objectives are clearly mentioned		
**3b-i) Bug fixes, Downtimes, Content Changes**		
**4a) CONSORT: Eligibility criteria for participants**		
The trial design is clearly stated in the manuscript		
**4a-i) Computer / Internet literacy**		
**4a-ii) Open vs. closed, web-based vs. face-to-face assessments:**		
Not applicable for the article		
**4a-iii) Information giving during recruitment**		
Face to face from a physician		
**4b) CONSORT: Settings and locations where the data were collected**		
Not applicable for the article		
**4b-i) Report if outcomes were (self-)assessed through online questionnaires**		
**4b-ii) Report how institutional affiliations are displayed**		
No online questionnaires will be passed		
**5) CONSORT: Describe the interventions for each group with sufficient details to allow replication, including how and when they were actually administered**		
**5-i) Mention names, credential, affiliations of the developers, sponsors, and owners**		
**5-ii) Describe the history/development process**		
The institut St Pierre is the only sponsor of the stdudy		
**5-iii) Revisions and updating**		
No application is used for this study		
**5-iv) Quality assurance methods**		
this is a research protocol		
**5-v) Ensure replicability by publishing the source code, and/or providing screenshots/screen-capture video, and/or providing flowcharts of the algorithms used**		
Assurance has been taken by the Institut		
**5-vi) Digital preservation**		
No app have been designed for this article		
**5-vii) Access**		
No app have been designed for this article		
**5-viii) Mode of delivery, features/functionalities/components of the intervention and comparator, and the theoretical framework**		
No app have been designed for this article		
**5-ix) Describe use parameters**		
No app have been designed for this article		
**5-x) Clarify the level of human involvement**		
No app have been designed for this article		
**5-xi) Report any prompts/reminders used**		
All measurements and interventions are realized by health practitioners		
**5-xii) Describe any co-interventions (incl. training/support)**		
No applicable for this article		
**6a) CONSORT: Completely defined pre-specified primary and secondary outcome measures, including how and when they were assessed**		
inclusion and exclusion criteria are clearly stated		
**6a-i) Online questionnaires: describe if they were validated for online use and apply CHERRIES items to describe how the questionnaires were designed/deployed**		

**6a-ii) Describe whether and how “use” (including intensity of use/dosage) was defined/measured/monitored**		
**6a-iii) Describe whether, how, and when qualitative feedback from participants was obtained**		
Based on other strength training papers		
**6b) CONSORT: Any changes to trial outcomes after the trial commenced, with reasons**		
data are not yet collected		
**7a) CONSORT: How sample size was determined**		
**7a-i) Describe whether and how expected attrition was taken into account when calculating the sample size**		
**7b) CONSORT: When applicable, explanation of any interim analyses and stopping guidelines**		
Gait parameters will be assessed with a 3D gait analysis , muscle strength will ba assessed with isokinetic dynamometer, spasticity with isokinetic dynamometer, and knee joint position sense will be assessed with isokinetic dynamometer. These variables will be evaluated at baseline (T0) and at the end of the intervention (T1) (3weeks after intervention)		
**8a) CONSORT: Method used to generate the random allocation sequence**		
No changes are yet to be reported		
**8b) CONSORT: Type of randomisation; details of any restriction (such as blocking and block size)**		
Not applicable for this research protocol		
**9) CONSORT: Mechanism used to implement the random allocation sequence (such as sequentially numbered containers), describing any steps taken to conceal the sequence until interventions were assigned**		
Not yet realized as this is a research protocol		
**10) CONSORT: Who generated the random allocation sequence, who enrolled participants, and who assigned participants to interventions**		
Not yet realized as this is a research protocol		
**11a) CONSORT: Blinding - If done, who was blinded after assignment to interventions (for example, participants, care providers, those assessing outcomes) and how**		
**11a-i) Specify who was blinded, and who wasn’t**		
**11a-ii) Discuss e.g., whether participants knew which intervention was the “intervention of interest” and which one was the “comparator”**		
Patient and practitioner will not be blinded		
**11b) CONSORT: If relevant, description of the similarity of interventions**		
Not yet realized as this is a research protocol		
**12a) CONSORT: Statistical methods used to compare groups for primary and secondary outcomes**		
Not yet realized as this is a research protocol		
**12a-i) Imputation techniques to deal with attrition / missing values**		
**12b) CONSORT: Methods for additional analyses, such as subgroup analyses and adjusted analyses**		
Only the isokinetic strengthening program change from one group to the other		
RESULTS		
**13a) CONSORT: For each group, the numbers of participants who were randomly assigned, received intended treatment, and were analysed for the primary outcome**		
Not yet realized as this is a research protocol		
**13b) CONSORT: For each group, losses and exclusions after randomisation, together with reasons**		
Not yet realized as this is a research protocol		
**13b-i) Attrition diagram**		
**14a) CONSORT: Dates defining the periods of recruitment and follow-up**		
Not yet realized as this is a research protocol		
**14a-i) Indicate if critical “secular events” fell into the study period**		
**14b) CONSORT: Why the trial ended or was stopped (early)**		
Not yet realized as this is a research protocol		
**15) CONSORT: A table showing baseline demographic and clinical characteristics for each group**		
Not yet realized as this is a research protocol		
**15-i) Report demographics associated with digital divide issues**		
**16a) CONSORT: For each group, number of participants (denominator) included in each analysis and whether the analysis was by original assigned groups**		
**16-i) Report multiple “denominators” and provide definitions**		
**16-ii) Primary analysis should be intent-to-treat**		
Not yet realized as this is a research protocol		
**17a) CONSORT: For each primary and secondary outcome, results for each group, and the estimated effect size and its precision (such as 95% confidence interval)**		
Not yet realized as this is a research protocol		
**17a-i) Presentation of process outcomes such as metrics of use and intensity of use**		
**17b) CONSORT: For binary outcomes, presentation of both absolute and relative effect sizes is recommended**		
Not yet realized as this is a research protocol		
**18) CONSORT: Results of any other analyses performed, including subgroup analyses and adjusted analyses, distinguishing pre-specified from exploratory**		
Not yet realized as this is a research protocol		
**18-i) Subgroup analysis of comparing only users**		
**19) CONSORT: All important harms or unintended effects in each group**		
Not yet realized as this is a research protocol		
**19-i) Include privacy breaches, technical problems**		
**19-ii) Include qualitative feedback from participants or observations from staff/researchers**		
Not yet realized as this is a research protocol		
DISCUSSION		
**20) CONSORT: Trial limitations, addressing sources of potential bias, imprecision, multiplicity of analyses**		
**20-i) Typical limitations in ehealth trials**		
**21) CONSORT: Generalisability (external validity, applicability) of the trial findings**		
**21-i) Generalizability to other populations**		
**21-ii) Discuss if there were elements in the RCT that would be different in a routine application setting**		
Not yet realized as this is a research protocol		

**22) CONSORT: Interpretation consistent with results, balancing benefits and harms, and considering other relevant evidence**		
**22-i) Restate study questions and summarize the answers suggested by the data, starting with primary outcomes and process outcomes (use)**		
**22-ii) Highlight unanswered new questions, suggest future research**		
Not yet realized as this is a research protocol		
Other information		
**23) CONSORT: Registration number and name of trial registry**		
Not yet realized as this is a research protocol		
**24) CONSORT: Where the full trial protocol can be accessed, if available**		
Not yet realized as this is a research protocol		
**25) CONSORT: Sources of funding and other support (such as supply of drugs), role of funders**		
Not yet realized as this is a research protocol		
**X26-i) Comment on ethics committee approval**		
**x26-ii) Outline informed consent procedures**		
The ethic consideration has been approved by the French national committee		
**X26-iii) Safety and security procedures**		
Not yet realized as this is a research protocol		
**X27-i) State the relation of the study team towards the system being evaluated**		
